# Imbalance of γδT17/γδTreg cells in the pathogenesis of allergic asthma induced by ovalbumin

**DOI:** 10.1590/1414-431X20187127

**Published:** 2018-07-10

**Authors:** Xia Yang, Jing-Hong Zhang, Wang-Sheng Deng, Chao-Qian Li

**Affiliations:** 1 Department of Emergency, the First Affiliated Hospital of Guangxi Medical University, The Guangxi Talent Highland for Emergency and Rescue Medicine, Nanning, Guangxi, China; 2 Department of Respiratory Medicine, Guangxi Vocational and Technical College of Health, Nanning, Guangxi, China

**Keywords:** Bronchial asthma, γδT17 cells, γδTreg cells, Airway inflammation

## Abstract

We aimed to explore the imbalance between the T helper 17 γδT cells (γδT17) and the regulatory γδT cells (γδTreg) in asthmatic mice. Male Balb/c mice were randomly divided into the normal control group and the asthmatic model group. The asthmatic model group mice were intraperitoneally injected with the mixture of ovalbumin (OVA)/Al(OH)_3_ and then activated by exposure of the animals to OVA atomization. Airway hyperresponsiveness (AHR) was determined by a non-invasive lung function machine. Hematoxylin and eosin and Alcian blue-periodic acid Schiff staining were done for histopathological analysis. Interleukin (IL)-17 and IL-35 levels in bronchoalveolar lavage fluid were detected by ELISA. The percentage of IL-17^+^ γδT cells and Foxp3^+^ γδT cells in spleen cells suspension were detected and the transcription levels of RORγt and Foxp3 in the lung tissue were determined. Compared with the normal control, the severity of airway inflammation and AHR were higher in the asthmatic mice. Furthermore, mice in the asthmatic group displayed significant increases of IL-17^+^ γδT cells, expression of IL-17A, and RORγt, whereas control mice displayed marked decreases of Foxp3^+^ γδT cells, expression of IL-35, and transcription factor Foxp3. In addition, the mRNA expression of RORγt was positively correlated with the percentage of IL-17^+^γδT cells, and the mRNA level of Foxp3 was positively correlated with the percentage of Foxp3^+^ γδT cells. The imbalance of γδT17/γδTreg in the asthmatic mice may contribute to the pathogenesis of OVA-induced asthma.

## Introduction

Bronchial asthma is a chronic inflammatory airway disease characterized by airway inflammation, airway hyper-responsiveness (AHR) and airway wall remodeling (1). Despite robust clinical studies and experimental animal models, the pathogenesis of asthma remains poorly defined. Some studies have indicated that the imbalance of T helper (Th) 1/Th 2 cells (Th1/Th2) immune pattern and Th17/regulatory T (Th17/Treg) dysregulation (2,3) may play a crucial role in the development and outcome of asthma; however, these imbalances cannot fully explain all the phenomena in asthma.

Emerging evidence has suggested that γδT cells, a T cell subset, have a critical role in the pathogenesis of asthma, such as airway inflammation and remodeling through the release of cytokines (4,5). Our previous studies have confirmed that γδT cells not only have a Th1/Th2 immune pattern (6), but also display a predominantly Th17 phenotype (7). It has previously been reported that γδT17 cells, as important proinflammatory cells that produced the IL-17 cytokine, were associated with the pathogenesis of various inflammation diseases, such as bronchial asthma, cancer, skin inflammation, and others (8–10). On the other hand, regulatory γδT cells (γδTreg) are the recently reported subset of γδT cells characterized by both expressions of TCRγδ and Foxp3, with potential immunosuppressive functions (11). Studies have shown the potential role of γδ regulatory T cells in inhibiting antitumor immune responses in patients diagnosed with multiple myeloma (12). However, studies on the relation of γδT17 cells and γδTreg cells in ovalbumin (OVA)-induced asthma are poorly reported.

Therefore, the relationship between γδT17 cells and γδTreg cells is postulated to be an important component in association with the pathogenesis of asthma. The aim of this study was to explore the imbalance of γδT17/γδTreg cells and related changes in cytokine expression in the pathogenesis of asthmatic mice and to further elucidate whether this imbalance is associated with alternation of inflammation in OVA-induced asthma.

## Material and Methods

### Experimental animals and protocols

Male Balb/c mice (4–6 weeks old, 18–22 g body weight) maintained under specific pathogen-free regions were obtained from the Laboratory Animal Center of Guangxi Medical University (Nanning, China). Room temperature and humidity were set at 23±3°C and 55.5±10%, respectively. All mice received a standard laboratory diet and water *ad libitum* and were maintained on a 12-h light-dark cycle. Animal experimental protocols were approved by the Ethical Principles in Animal Research adopted by the Guangxi Medical University for Animal Experimentation.

### Experimental models

**Figure 1.**
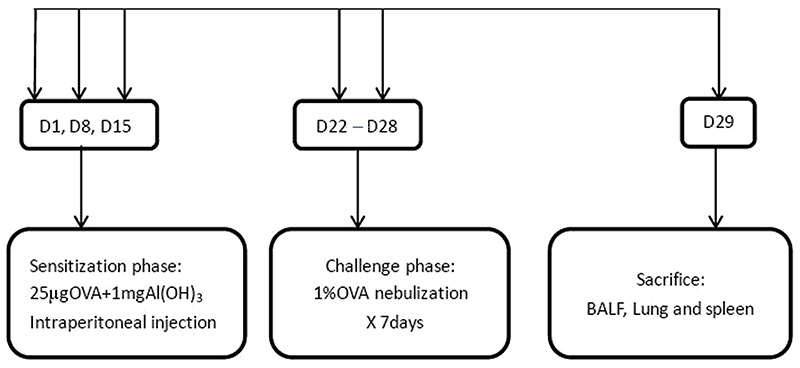
Flow chart of the ovalbumin (OVA) sensitization model of asthmatic BALB/c mice. The mice were randomly divided into 2 groups and treated as shown above. D: day.

Experimental animals were randomly divided into two groups: normal control and asthmatic model, with 6 mice in each group. The normal control group was treated with saline at the sensitization and challenge stages. The asthmatic model group was sensitized and challenged with OVA to establish the asthmatic model, according to methods proposed by our previous report with minor modifications (13) (Figure 1). Mice were sensitized with 25 μg of OVA (grade V, Sigma-Aldrich, USA) emulsified in 1 mg of aluminum hydroxide (Chengdu Kelong Chemical Reagent Factory, China) in a total volume of 200 μL on days 1, 8, and 15 by intraperitoneal administration. Beginning on the 22nd day, the mice were challenged with 1% OVA (mg/mL) for 30 min per day by an ultrasonic nebulizer (WH-2000, China) in a closed chamber for 7 days to establish models.

### Airway responsiveness

AHR was assessed using a double-chamber plethysmography device (TBL4500, Buxco, USA) based on the increase in the specific airway resistance (sRaw). In brief, the mice were exposed to nebulized PBS for 3 min to establish baseline sRaw values, followed by exposure to increasing concentrations of nebulized methacholine (6.25–25 mg/mL; Sigma, USA) using an Aerosonic ultrasonic nebulizer. Following each nebulization cycle, recordings were obtained for 3 min. The sRaw values measured during each 3-min sequence were averaged and reported for each methacholine concentration. The increase in sRaw was calculated as follows: sRaw with each methacholine concentration - sRaw with PBS) / sRaw with PBS.

### Sample collection and processing

Twenty-four hours after the last challenge, all animals were anesthetized with pentobarbital. Specimens of bronchoalveolar lavage fluid (BALF), lung, and spleen were harvested. BALF (1200 μL) was collected as previously described (14), centrifuged at 1500 *g* for 10 min at 4°C, and the supernatant was immediately frozen at −80°C for measurement of cytokine levels.

In order to obtain spleen cell suspensions, spleens were removed, cut into small pieces, ground gently into single-cell particles, and filtered through nylon mesh. The cell suspension was centrifuged at 300 *g* for 10 min at 4°C. After that, erythrocytes were removed as described previously, and the spleen cell pellets were washed twice with cold PBS.

The right lung tissue was quick-frozen by immersion in liquid nitrogen, and then stored until quantitative real-time PCR was performed.

### Histology and morphometry assay

The left lung tissue was fixed with 4% paraformaldehyde, embedded in paraffin, cut sagittally into 4-μm sections, and stained with hematoxylin and eosin (H&E) and Alcian blue-periodic acid Schiff (AB-PAS) for histological analysis. The micro-sections were stained for the examination of inflammation and mucus production under a microscopic observation (Olympus, Japan). For each animal, 10 fields at a magnification of 200× were measured randomly from HE staining and were measured randomly from AB-PAS staining (400×). Two investigators independently measured the origin of stained tissues in a blinded manner.

### Cytokine measurement

The concentrations of IL-17 and IL-35 in BALF of OVA-induced asthmatic mice were measured by commercial ELISA kits according to the manufacturer's instructions (Cusabio Biotech, China). ELISA kit of IL-17 detects from 47 pg/mL to 3000 pg/mL, and ELISA kit of IL-35 detects from 15.6 pg/mL to 1000 pg/mL. The absorbance was measured at 450 nm by a micro-plate ELISA reader (BioTek, USA). All samples were assayed in duplicate.

### Flow cytometry

The expression markers on T cells from spleen were determined by flow cytometry using the following antibodies: Percp-CD3, APC-γδT, PE-IL-17, and PE-Foxp3 purchased from BD Pharmingen (USA) or eBioscience (USA). Cell surface staining was performed according to the standard procedures. For intracellular detection of cytokines, cells were stimulated with phorbol-myristate-acetate (25 ng/mL; Sigma-Aldrich) and ionomycin (1 ng/mL; Sigma-Aldrich) in the presence of GolgiPlug™ (BD Pharmingen) for 4 h at 37°C in 5% CO_2_. The cells were then washed and stained with fluorescent antibodies against CD3 at room temperature in the dark. After surface staining, cells were fixed/permeabilized in fixation/permeabilization solution (Cytofix/Cytoperm™; BD Pharmingen) according to the manufacturer's protocol, and stained with anti-IL-17 mAbs/anti-Foxp3-mAbs for 30 min at 4°C. Cells were then washed with Perm/Wash Buffer (BD Pharmingen) and resuspended in PBS +2% FBS for flow cytometry analysis. Flow cytometry was performed on a BD FACS Canto II (BD Biosciences, USA) and analyzed using FCS Express 4 software (De Novo Software, USA).

### Real-time quantitative PCR

For quantifying the transcription levels of RORγt and Foxp3, total RNA (1 μg) was extracted from the right lung tissue with TRIzol (Invitrogen, Life Technologies, USA) according to the manufacturer's instructions. Complementary DNA (cDNA) was prepared using oligo(dT) primers (PrimeScript™RT, reagent Kit, Takala, Japan). Quantitative RT-PCR was performed by duplicate with SYBR Green I (SYBR¯Premix Ex Taq™, Takala) using an Applied Biosystems 7500 (ThermoFisher Scientific, USA) according to the manufacturer's instructions. DNA was amplified under the following conditions: denaturation at 95°C for 30 s, extension at 95°C for 5 s, 60°C for 34 s, and the samples were amplified for 40 cycles. The following primers were used: 5′-AATTCCATCATGAAGTGTGA-3′, 5′-ACTCCTGCTTGCTGATCCAC-3′ for β-actin; 5′- ACggCCCTggTTCTCATCA -3′ and 5′- CCAAATTgTATTgCAgATgTTCCAC -3′ for RORγt; 5′-AGTTCCTTCCCAGAGTTCTTCCA-3′ and 5′-GCTCAGGTTGTGGCGGATG-3′ for Foxp3. Mouse β-actin was used as an internal control, and levels of each gene were normalized to β-actin expression using the delta-delta Ct method. The identity of the amplified products was examined using agarose gel electrophoresis and melt curve analysis.

### Statistical analysis

Data are reported as means±SD. Differences between groups were compared with unpaired Student's *t*-test. Correlations between variables were determined by Spearman's rank correlation test. Analysis was completed using SPSS version 19.0 statistical software (USA), and P<0.05 was considered to indicate statistical significance.

## Results

### Symptoms of the asthmatic mice

No mice died before sacrifice. During the challenge phase, OVA-induced asthmatic mice were found with different degrees of shortness of breath, cyanosis of lips, reduction of spontaneous activities, loss of weight, and incontinence, whereas mice in the control group displayed no manifestations.

### Airway resistance

**Figure 2.**
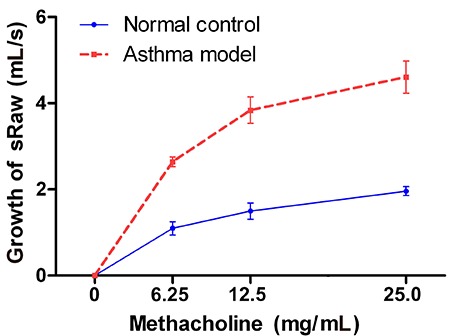
Change in specific airway resistance (sRaw) of mice after being sensitized and challenged with ovalbumin and inhalation of different concentrations of methacholine compared with the control group (P<0.05). Data are reported as means±SD (Student's *t*-test).

Compared with the normal control group, sRaw showed a significant increase at each concentration of methacholine in the asthmatic model group (P<0.05, Figure 2).

### Histology and morphometry assay

**Figure 3.**
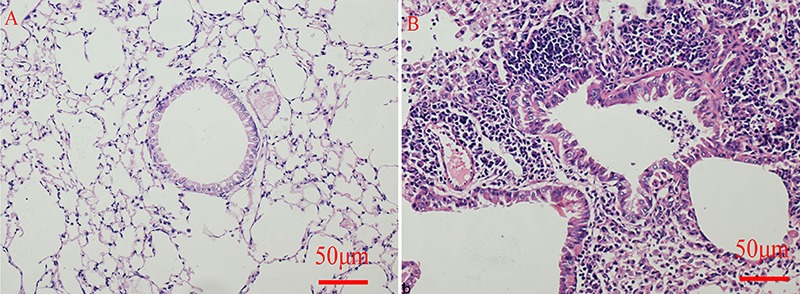
HE staining (×200; bar, 50 μm) showing the asthmatic group (*B*) with spotty infiltration of eosinophils around the airway, bronchioles, and blood vessels, compared to the normal control group (*A*).

**Figure 4.**
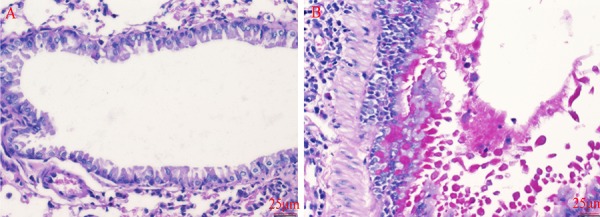
Alcian blue-periodic acid Schiff staining (×400; bar, 25 μm) showing the normal control group with rare goblet cell and no mucus secretion (*A*) and the asthmatic model group (*B*) with increased goblet cell hyperplasia in airway epithelia and mucus secretion.

From the H&E staining, the normal control group showed normal tissue, with no inflammatory cells. The asthmatic model group showed a significant infiltration of inflammatory cells around the airway, bronchioles, and blood vessels; inflammatory cells were mainly eosinophils and lymphocytes (Figure 3A and B). From the AB-PAS staining, the normal control group showed rare goblet cells and no mucus secretion, and the asthmatic model group had more goblet cell hyperplasia in airway epithelia and more mucus secretion (Figure 4A and B). The above results demonstrated that OVA administration successfully induced asthma.

### Cytokines analysis

**Figure 5.**
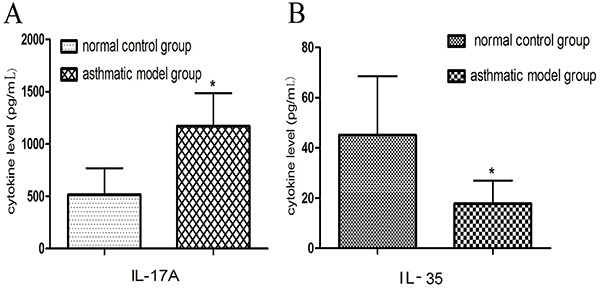
Concentrations of interleukin (IL)-17A (*A*) and IL-35 (*B*) in bronchoalveolar lavage fluid (BALF) were examined by ELISA using specific cytokine detection kits. Data are reported as means±SD. *P<0.05 *vs* the normal control group (*t*-test)

As shown in Figure 5, IL-17 expression was significantly higher in the asthmatic model group than in the normal control group (control group: 515.5±254.3 pg/mL, asthmatic group: 1171.2± 315.1 pg/mL, P=0.003). However, IL-35 expression was significantly decreased in the asthmatic model group, compared with the normal control group (control group: 45.1±23.4 pg/mL, asthmatic group: 17.7±9.2 pg/mL, P=0.024). These data indicated that changes in the expression of IL-17 and IL-35 may contribute to the γδT17 and γδTreg imbalance.

### Percentage of γδT17 and γδTreg cells

**Figure 6.**
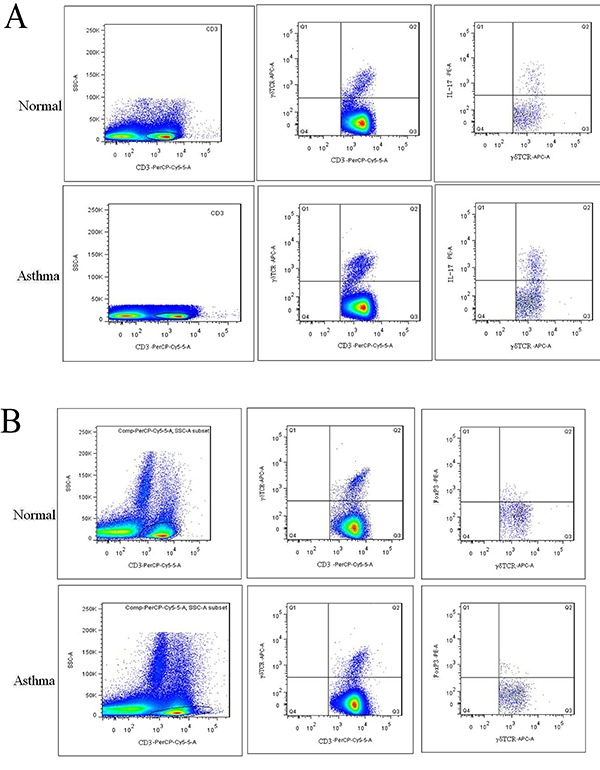
Spleens from ovalbumin-treated mice and the normal control group were analyzed to determine the percentage of γδT17 cells (*A*) and γδTreg cells (*B*) among the CD3^+^ T cells by flow cytometry.

**Figure 7.**
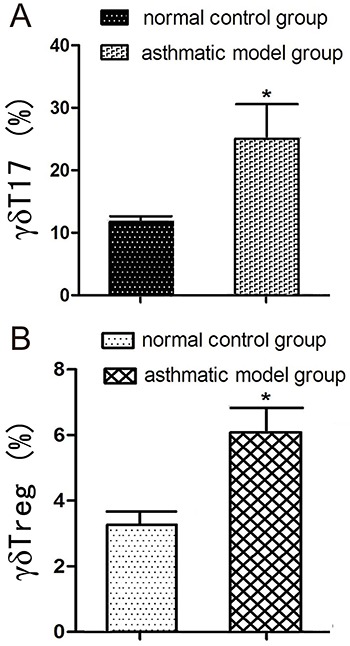
Comparative analysis of γδT17 cells (*A*) and γδTreg cells (*B*). Data are reported as means± SD (n=6). *P<0.05 *vs* normal control group (*t*-test).

As shown in Figures 6 and 7, the asthmatic model group exhibited an increase in γδT17 cells (25.0±5.5%) compared with the normal control group (11.7±0.8%). On the other hand, the normal control group exhibited a population of γδTreg cells of 6.0±0.8%. Compared with the normal control group, OVA-induced mice showed a significantly lower proportion of γδTreg cells (3.2±0.4%). These findings indicated that OVA could affect the percentage of γδT17 and γδTreg cells in the spleen of animals.

### mRNA expression of RORγt and Foxp3

**Figure 8.**
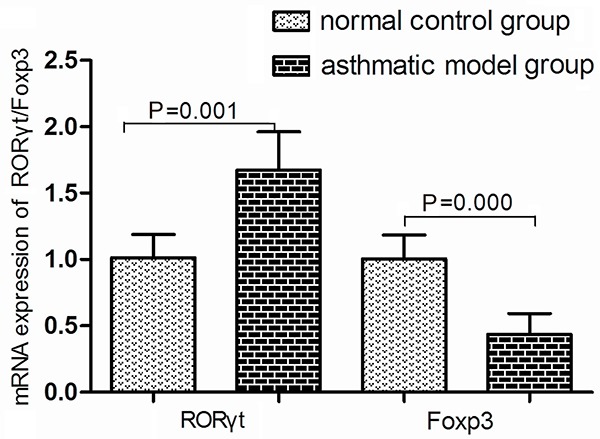
BALB/c mice were sensitized and challenged with ovalbumin. mRNA expression of RORγt and Foxp3 in lung tissues was determined by real-time PCR analysis. Data are reported as means±SD (*t*-test).

As shown in Figure 8, compared with the normal control group, the mRNA expression of RORγt was significantly increased in the asthmatic model groups. Conversely, the mRNA expression Foxp3 decreased in the model group after OVA administration compared with the normal control group.

### Correlation between mRNA expressions and γδT17 and γδTreg

**Figure 9.**
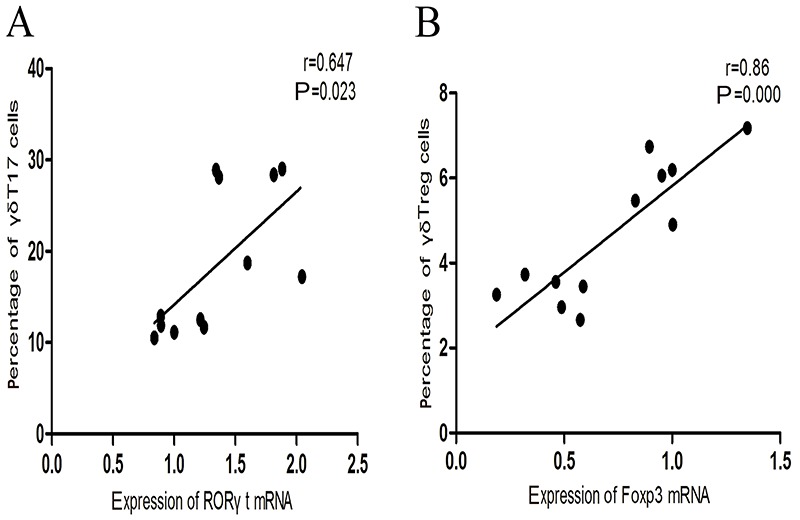
Correlation between (*A*) mRNA of RORγt and the percentage of γδT17 cells (r=0.647, P<0.05), as well as between Foxp3 and the percentage of γδTreg cells (r=0.86, P<0.05) in the asthmatic model group (*B*).

As shown in Figure 9, there were positive correlations between the mRNA of RORγt and the percentage of γδT17 cells (r=0.907, P<0.05), as well as between Foxp3 and the percentage of γδTreg cells (r=0.86, P<0.05) in the asthmatic model group (Figure 9A and B). It appears that RORγt and Foxp3 expression were closely related to the proportion of γδT17 and γδTreg cells in the lungs of mice with OVA-associated bronchial asthma.

## Discussion

Asthma is a chronic lung disease of which the mechanism is little known. As human experiments are impossible, animal experiments play an important role in the understanding of the mechanism of asthma. The ideal evaluation index of the asthmatic mouse model is AHR and airway inflammation (15). To determine the effects of OVA on the balance between γδT17 and γδTreg cells, we used classical methods to prepare murine models of asthma. The findings of our study were in accordance with the study of Da-zhen Wei and colleagues (16). Taken together, our results confirm that we established a successfully asthmatic model.

Evidence has emerged indicating that the pathogenesis of asthma is mainly related with dysregulated immune responses by Th1/Th2 cells and Th17/Treg cells (17). However, with the discovery of T cells, its mechanism is constantly changing. Recent studies have indicated γδT cells play a crucial role in the occurrence and development of autoimmune diseases and pathogen-induced immune responses, but the specific mechanisms need further investigation (18).

Emerging evidence suggests that Th17/Treg imbalance contributes to the development of asthma (19), showing an increase of Th17 cells and suppression of Treg development. Th17 cells have a role in chronic inflammation. As a subset of γδT cells, γδT17 cells are regarded as having a role in immune inflammatory diseases that are characterized by the secretion of IL-17A and the release of a large number of downstream inflammatory factors (19,20). On the other hand, studies have already shown that Foxp3^+^γδT cells, also known as γδTregs, were involved in some diseases, which may inhibit the activation and function of T cells involved in antigen-specific immune responses (21).

In the present study, we investigated dynamic changes in γδT17 and γδTregs percentages and analyzed the potential effects of their imbalance on the development and outcomes of asthmatic mice. Our data showed significantly increased percentages of IL-17^+^γδT cells and decreased Foxp3^+^γδT cells in the spleen cells suspension of the asthmatic mice compared to the control mice. We further found that γδT1-related cytokine IL-17A was increased in the asthmatic mice, which is consistent with the higher percentage of IL-17^+^γδT cells. The expression of IL-35 and the percentage of γδTreg cells also followed the above change. Moreover, evidence suggests that γδT17 cells are associated with the levels of RORγt, and γδTregs are characterized by the high expression of forkhead/winged helix family transcriptional repressor p3 (Foxp3) (22,23)_ENREF_18. Based on the findings mentioned above, the mRNA expression of RORγt and Foxp3 was observed in the lung tissue, showing the following changes: the mRNA expression of RORγt was significantly higher in the asthmatic model group than in the normal control group, whereas the mRNA expression of Foxp3 was decreased in the asthmatic mice. More importantly, there were positive correlations between the mRNA of RORγt and the percentage of γδT17 cells, as well as between Foxp3 and the percentage of γδTreg cells in the asthmatic model group. It appears that RORγt and Foxp3 expressions were closely related to the proportion of γδT17 and γδTreg cells in the lungs of mice with OVA-associated asthma.

Consequently, the above results demonstrated that the relationship between γδT17 and γδTreg cells may be involved in the pathogenesis of asthma induced by OVA. There is a possibility that the γδT17-related immune reaction became unbalanced although more γδT17 cells were involved. In contrast, γδTreg cells frequency was significantly negatively associated with the process. Further studies may lead to the effects of immunotherapy on asthma.
